# Gut microbial composition in patients with atrial fibrillation: effects of diet and drugs

**DOI:** 10.1007/s00380-020-01669-y

**Published:** 2020-07-18

**Authors:** Tokiko Tabata, Tomoya Yamashita, Koji Hosomi, Jonguk Park, Tomohiro Hayashi, Naofumi Yoshida, Yoshihiro Saito, Koji Fukuzawa, Kana Konishi, Haruka Murakami, Hitoshi Kawashima, Kenji Mizuguchi, Motohiko Miyachi, Jun Kunisawa, Ken-ichi Hirata

**Affiliations:** 1 Division of Cardiovascular Medicine, Department of Internal Medicine, Kobe University Graduate School of Medicine, 7-5-1 Kusunoki-cho, Chuo-ku, Kobe, 6500017 Japan; 2 Laboratory of Vaccine Materials, Center for Vaccine and Adjuvant Research, and Laboratory of Gut Environmental System, National Institutes of Biomedical Innovation, Health and Nutrition (NIBIOHN), Osaka, Japan; 3 Laboratory of Bioinformatics, National Institutes of Biomedical Innovation, Health and Nutrition (NIBIOHN), Osaka, Japan; 4 Department of Physical Activity Research, National Institutes of Biomedical Innovation, Health and Nutrition (NIBIOHN), Tokyo, Japan; 5 Institute for Protein Research, Osaka University, Osaka, Japan

**Keywords:** Atrial fibrillation, Gut microbiota, Dietary habits, n-3 polyunsaturated fatty acids, Eicosadienoic acid

## Abstract

Atrial fibrillation (AF) reduces the quality of life by triggering stroke and heart failure. The association between AF onset and gut metabolites suggests a causal relationship between AF and gut microbiota dysbiosis; however, the relationship remains poorly understood. We prospectively enrolled 34 hospitalized patients with AF and 66 age-, sex-, and comorbidity-matched control subjects without a history of AF. Gut microbial compositions were evaluated by amplicon sequencing targeting the 16S ribosomal RNA gene. We assessed differences in dietary habits by using a brief-type self-administered diet history questionnaire (BDHQ). Gut microbial richness was lower in AF patients, although the diversity of gut microbiota did not differ between the two groups. At the genus level, *Enterobacter* was depleted, while *Parabacteroides*, *Lachnoclostridium*, *Streptococcus,* and *Alistipes* were enriched in AF patients compared to control subjects. The BDHQ revealed that the intake of n-3 polyunsaturated fatty acids and eicosadienoic acid was higher in AF patients. Our results suggested that AF patients had altered gut microbial composition in connection with dietary habits.

**Electronic supplementary material:**

The online version of this article (10.1007/s00380-020-01669-y) contains supplementary material, which is available to authorized users.

## Introduction

There is extensive evidence that gut microbiota has important functions in host metabolism and immunity. Gut microbiota and its metabolites are known to play a role in the pathogenesis of metabolic disease [1] and chronic inflammatory disease [2], and their association with other diseases has been discovered, including the association between gut microbiota and cardiovascular diseases [3, 4]. We previously reported that amounts of *Bacteroides vulgatus* and *Bacteroides dorei—*predominant gram-negative gut microbes—were lower in patients with coronary artery disease (CAD) compared to control subjects [5]. Administration of these two *Bacteroides* species inhibited atherogenesis in apoe^−/−^ mice via decreasing inflammation. The gut microbiota-derived metabolites of dietary choline or carnitine, trimethylamine (TMA), and trimethylamine-*N*-oxide (TMAO) are related to cardiovascular disease, including atrial fibrillation (AF) [6–8]. In an earlier study, we investigated gut microbial composition and host plasma metabolites in heart failure and demonstrated the alteration of gut microbial composition and gain of plasma TMAO [9].

AF is the most common arrhythmia that increases the risk of heart failure, cerebral infarct, and arterial embolism. In the current aging society, patients with AF are increasing [10]. Obesity, type 2 diabetes mellitus, and hypertension are reportedly associated with gut microbial dysbiosis [1, 11] and are independent risk factors of AF. The gut microbial metabolites lipopolysaccharide (LPS), TMAO, and indoxyl sulfate (IS) increase the instability of atrial electrophysiology [8, 12, 13] and are associated with AF [14–16].

Several factors, including age, ethnicity, residential location, and dietary habits, affect gut microbial composition. Diet also influences host metabolism, e.g., high-fat diet induces insulin resistance via alteration of gut microbial composition and metabolic endotoxemia [17]. The Prevención con Dieta Mediterránea (PREDIMED) trial demonstrated that extra-virgin olive oil in a Mediterranean diet may reduce AF risk [18]. Low-carbohydrate diets were associated with increased risk of AF [19]. In AF patients, plasma LPS levels predicted major cardiovascular events and were negatively affected by adherence to a Mediterranean diet [14].

A recent investigation [20] of gut microbial composition in AF patients revealed differences in gut microbiota relative to control subjects with different comorbidities and medications. Alteration of gut microbiota in AF patients remains poorly understood, much less the association between gut microbiota and dietary habits. The present study reports an analysis of gut microbiota and dietary habits in AF patients.

## Materials and methods

### Study population

Between January 2018 and September 2018, we enrolled 50 consecutive patients who were admitted to Kobe University Hospital for catheter ablation against AF. However, 16 patients were excluded because of the following reasons: two patients withdrew from the study, 11 patients were not able to collect fecal samples, and three patients did not have less than moderate valvular disease or left ventricular dysfunction. Therefore, samples from 34 patients were finally obtained for analysis. Patients provided written informed consent upon study enrollment. The study was conducted according to the guidelines of the Declaration of Helsinki, was approved by the Ethics Committee of Kobe University (approval No. 180140), and was registered with the UMIN Clinical Trials Registry (trial registration no. UMIN000027975).

The AF group included patients having their first to third session of catheter ablation and previously documented disease on 12-lead electrocardiography or Holter electrocardiography. Evaluation included physical examination, blood chemistry, 12-lead electrocardiography, chest radiography, and echocardiography. Patients with hepatic diseases, such as viral hepatitis and liver cirrhosis, renal failure (serum creatinine levels of > 2.0 mg/dl), collagen disease, malignancy, active infectious diseases, inflammatory intestinal diseases, moderate and severe organic heart disease, and patients with antibiotic or steroid treatment within 2 weeks prior to admission were excluded. Hypertension, diabetes mellitus, and dyslipidemia were recorded according to relevant guidelines [21].

We extracted 66 age-, sex-, and comorbidity-matched control subjects from a healthy Japanese cohort developed by the National Institutes of Biomedical Innovation, Health and Nutrition (NIBIOHN). The presence of hypertension, diabetes mellitus, and dyslipidemia, and the absence of AF were based on a self-assessment questionnaire. All experiments in NIBIOHN were approved by the Ethics Committee of NIBIOHN (part of the control cohort was registered as UMIN Clinical Trial Registry UMIN000023270) and were conducted in accordance with the guidelines of the Declaration of Helsinki, together with informed consent from all participants.

### Fecal sample collection and bacterial DNA extraction

Fecal samples of AF patients were collected during hospitalization, while fecal samples of control subjects were collected at home. Samples were placed in 15 ml vials containing 3 ml guanidine thiocyanate (GuSCN) solution (TechonoSuruga Laboratory Co., Ltd., Shizuoka, Japan), mixed by vortexing, and refrigerated at 4 °C. DNA was extracted from samples in GuSCN solution by the beating method [22].

### 16S ribosomal RNA gene amplification and sequencing

Bacterial DNA from the fecal samples was amplified by PCR. The V3-V4 regions of the bacterial 16S rRNA gene were amplified using the following primers: forward, 5′-TCGTCGGCAGCGTCAGATGTGTATAAGCGACAGCCTACGGGNGGCWGCAG-3′, and reverse, 5′-GTCTCGTGGGCTCGGAGATGTGTATAAGAGACAGGACTACHVGGGTATCTAATCC-3′. A detailed description of the primer set and PCR conditions is available elsewhere [22]. After addition of the sequencing adapters, the amplicons were sequenced using the Illumina MiSeq platform (Illumina Inc., San Diego, CA, USA) according to the manufacturer’s instructions.

Obtained paired-end FASTQ files from MiSeq were trimmed and merged before operational taxonomic units (OTUs) were picked. OTU classification and diversity analysis were performed using the QIIME pipeline (v 1.9.1) [23]. All steps from FASTQ file trimming to gut microbiota diversity analysis were automatically performed according to previously described methods [24]. The OTUs were clustered against the SILVA 128 reference database [25] at 97% similarity using the USEARCH algorithm [26]. Taxonomic classification was performed by using the SILVA 128 reference database. Clustering analysis was performed using the R package pvclust based on Pearson’s correlation coefficient and the ward.D2 method (nboost = 10,000).

### Dietary habit questionnaire

Dietary habits during the preceding month were assessed with a brief-type self-administered diet history questionnaire (BDHQ) [27]. Daily intake estimates for food, energy, and selected nutrients were calculated using an ad hoc computer algorithm developed for the BDHQ, based on the Standard Table of Food Composition in Japan [28].

### Statistical analysis

Statistical analyses were performed using R software, version 3.1.0 (https://www.r-project.org/), JMP version 10 (SAS Institute, Cary, NC), and Prism version 7.0 (GraphPad Software, San Diego, CA, USA). The Shapiro–Wilk test was used to check for normal distribution. Normally distributed data are expressed as mean ± SD or SEM and non-normally distributed as median ± interquartile range (25th–75th percentiles. Two-tailed Student’s *t* test was used to compare normally distributed data and the Mann–Whitney *U* test for non-normally distributed data. Fisher’s exact test or *χ*^2^ test was used to compare categorical variables. For all tests, *p* < 0.05 indicated statistical significance. We analyzed gut microbiomes exhibiting at least 0.1% abundance in all samples to determine between-group differences in composition. The Shannon–Wiener index, and Chao1 index were calculated using R software. Principal component analysis was performed using JMP.

## Results

### Baseline patient characteristics

Baseline vital signs and medications of the two groups are shown in Table 1. In contrast to a previous report [20], we controlled as many confounding factors in the control subjects as possible. The groups were similarly distributed in terms of age; sex; body mass index; and comorbidities, including hypertension, diabetes mellitus, and dyslipidemia. Medication matching between the two groups was difficult. Anticoagulant, proton-pump inhibitor (PPI), beta blocker, and antiarrhythmic drug use were higher in AF patients than in control subjects.

**Table 1:** Characteristics of control subjects vs. atrial fibrillation patients

	Control subjects (*n* = 66)	AF patients (*n* = 34)	*p* value
Age (years)	65.3 ± 0.9	65.6 ± 1.4	0.88
Male, sex	50 (77.0%)	26 (76.5%)	> 0.99
Body mass index (kg/m^2^)	23.6 (21.5–26.8)	24.8 (22.6–27.2)	0.25
Atrial fibrillation			
Paroxysmal	–	19 (55.9%)	
Persistent	–	15 (44.1%)	
Vitals			
SBP (mmHg)	130.5 (130.3–139.4)	119.0 (116.3–126.0)	< 0.01
DBP (mmHg)	80.7 ± 1.4	69.4 ± 1.9	< 0.01
Comorbidity			
Hypertension	45 (68.2%)	23 (67.6%)	> 0.99
Diabetes mellitus	16 (24.2%)	7 (20.6%)	0.80
Dyslipidemia	43 (65.2%)	21 (61.8%)	0.83
Medication			
ARB and/or ACEi	32 (48.5%)	14 (41.2%)	0.53
β-Blocker	6 (9.1%)	11 (32.4%)	< 0.01
Calcium-channel blocker	23 (34.9%)	15 (41.2%)	0.27
Diuretics (loop or thiazide)	3 (4.5%)	1 (2.9%)	> 0.99
Statin	23 (34.8%)	8 (23.6%)	0.27
PPI/H2 blocker	8 (12.1%)	28 (82.4%)	< 0.01
Antiarrhythmic	0 (0%)	11 (33.8%)	< 0.01
Anticoagulant agent	1 (1.5%)	34 (100%)	< 0.01

Binary data are presented as number (%). Values are expressed as mean ± standard deviation for normally distributed variables, and median and interquartile range for non-normally distributed variables

*ACEi* angiotensin-converting enzyme inhibitor, *ARB* angiotensin II receptor blocker, *DBP* diastolic blood pressure, *PPI* proton-pump inhibitor, *SBP* systolic blood pressure

### Gut microbial composition in AF patients and control subjects

**Fig. 1:**
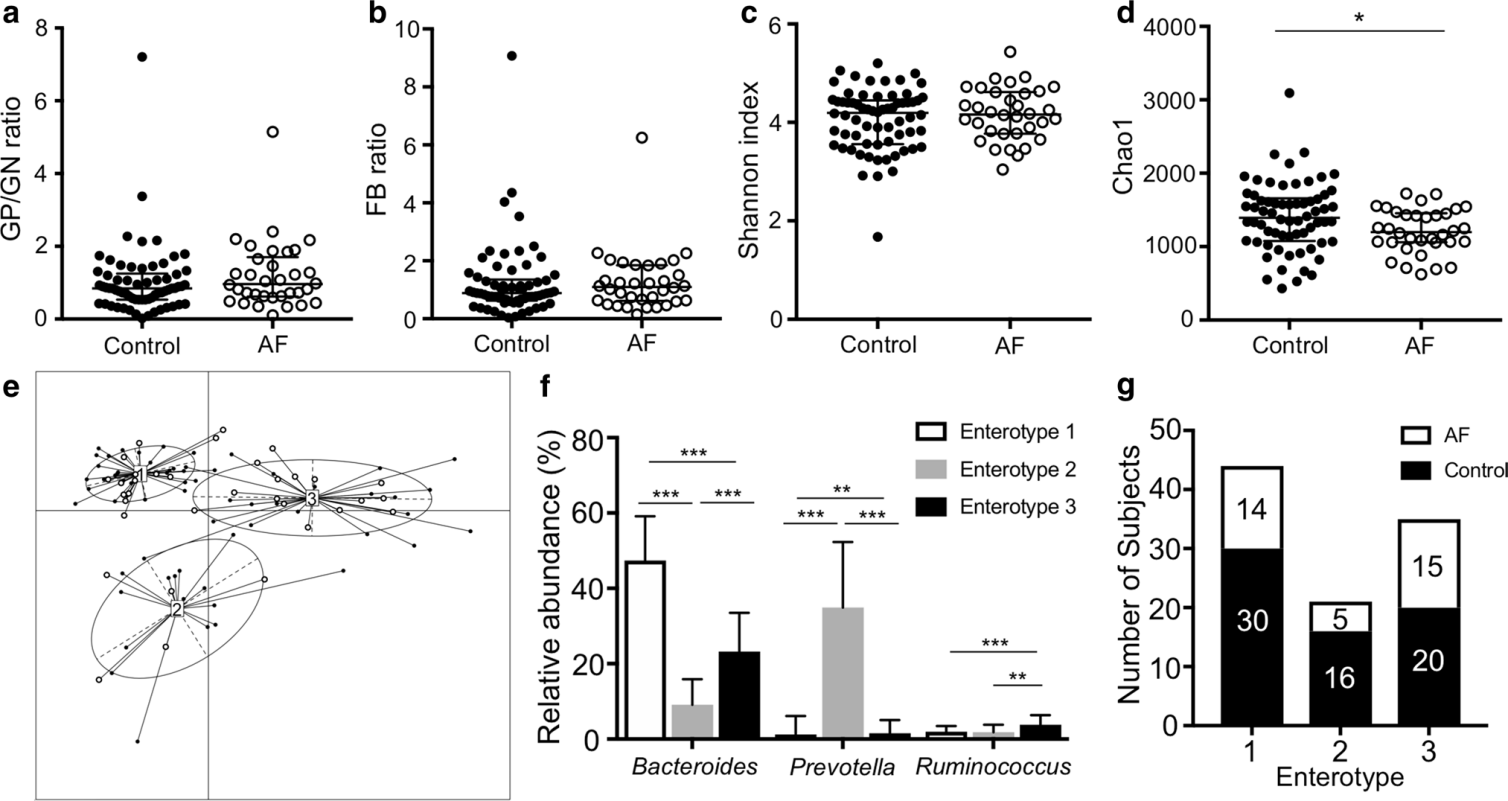
Gut microbial features in control subjects and AF patients. **a** Ratio of GP to GN strains. **b** Ratio of *Firmicutes* to *Bacteroidetes* (FB). **c**, **d** α-Diversity of gut microbiota. **e** Participants were classified into three enterotypes based on genus level abundance in gut microbiota. **f** Abundances of the main contributors of each enterotype. **g** Distribution of control subjects and AF patients in each enterotype. *p < 0.05; **p < 0.01; ***p < 0.001. *GP* gram-positive, *GN* gram-negative, *FB* ratio of *Firmicutes* to *Bacteroidetes*

There were no significant differences in the gram-positive/gram-negative bacteria ratio (GP/GN ratio: control 0.85 [0.54–1.25] vs. AF 0.96 [0.62–1.70], *p* = 0.21) or the *Firmicutes*/*Bacteroides* ratio (F/B ratio: control 0.88 [0.62–1.35] vs. AF 1.10 [0.62–1.84], *p* = 0.48) between the two groups (Fig. 1a, b). Gut microbial diversity and richness were measured by the Shannon index and Chao1, respectively. The Shannon index, which expresses diversity, including the evenness of gut microbiota, was comparable between both groups. (Shannon index: control 4.2 [3.6–4.4] vs. AF 4.2 [3.8–4.6], *p* = 0.56; Fig. 1c). Conversely, Chao1, which expresses the richness of gut microbiota, was lower in AF patients than in control subjects (Chao1: control 1392 [1078–1660] vs. AF 1200 [1059–1454], *p* = 0.03, Fig. 1d).

Gut microbial profiles were broadly categorized into three enterotypes at the genus level, according to a published procedure (Fig. 1e) [5, 29]. Each enterotype was characterized by a high abundance of a specific genus as follows: *Bacteroides* in enterotype 1, *Prevotella* in enterotype 2, and *Ruminococcus* in enterotype 3 (Fig. 1f). AF patients were more likely to be categorized into enterotype 3 than the control subjects (Fig. 1g).

**Fig. 2:**
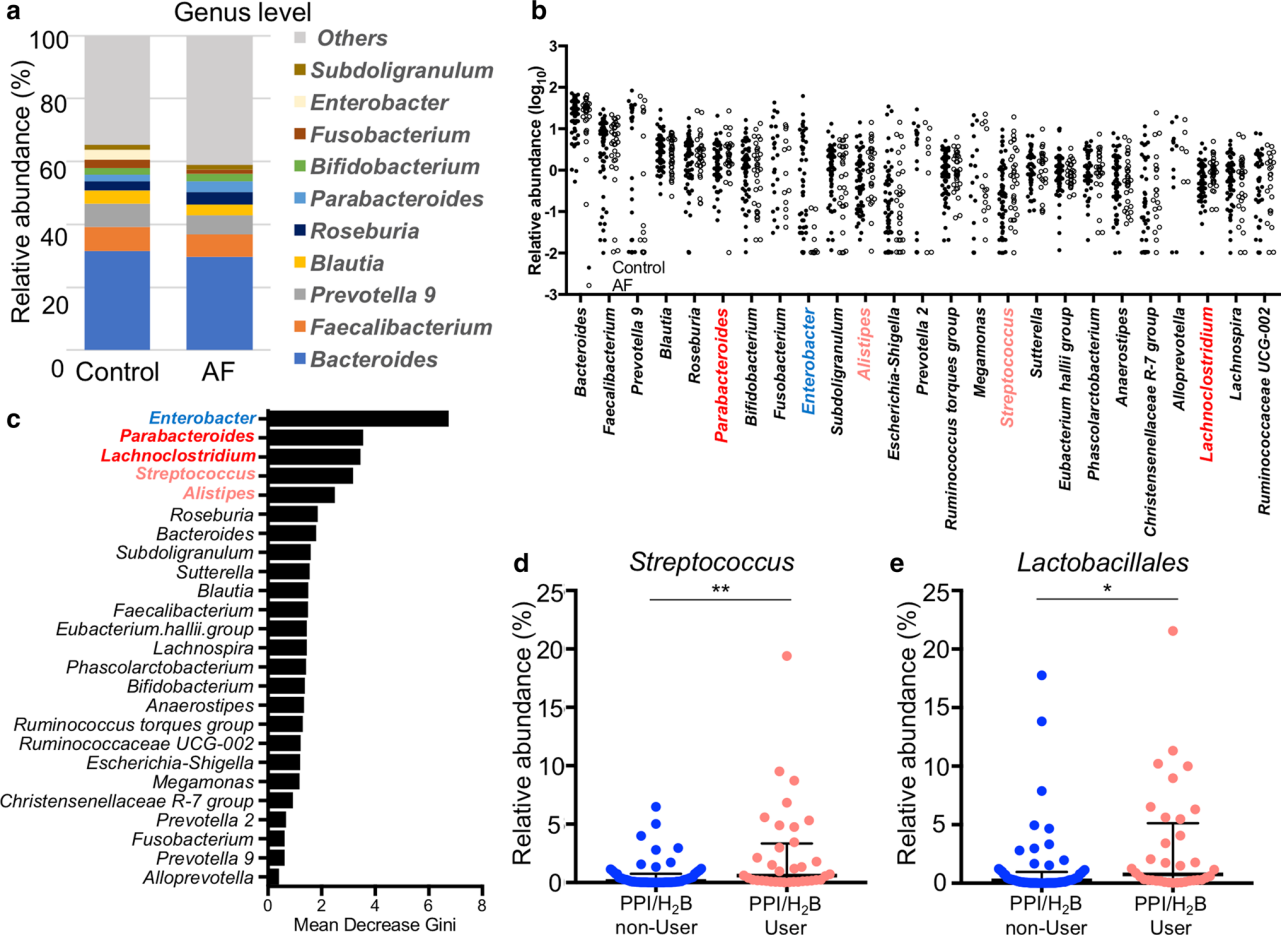
Gut microbial composition in control subjects and AF patients. **a** Mean abundance of genus level. **b** Relative abundance (log10) plot of the top 25 most abundant genera. The genus names are colored according to the *p* value between AF patients and control subjects as red or blue (*p* < 0.01), light red (*p* < 0.05), and black (*p* ≥ 0.05). **c** The ranking of contribution to AF by random forest analysis against the top 25 most abundant genera. **d**, **e** Comparative analyses of PPI-associated genera and orders between PPI/H2B blocker users and non-users. *p < 0.05; **p < 0.01. *AF* atrial fibrillation, *PPI* proton-pump inhibitor

We performed 16S rRNA sequencing of samples and analyzed the abundance of 16S reads at the genus level. The mean relative abundance of the genus level is shown in Fig. 2a. The relative abundance of the 25 most abundant genera is shown in Fig. 2b. The genus *Enterobacter* was significantly lower, whereas *Parabacteroides*, *Lachnoclostridium*, *Streptococcus*, and *Alistipes* were significantly higher in AF patients than in control subjects. To confirm the gut microbial contribution to the occurrence of AF, random forest analysis of the 25 most abundant genera was performed. The genera *Enterobacter*, *Parabacteroides*, *Lachnoclostridium*, *Streptococcus,* and *Alistipes* ranked the highest (Fig. 2c). Microbiome data at phylum and genus levels are shown in Supplemental Tables 1 and 2. Although by a small amount, the genera *Butyricimonas* and *Dorea* were increased in AF patients (Supplemental Table 2).

Figure 2d, e reveal the proportion of PPI-associated genera and orders in PPI/H2 blocker non-users vs. users. As previously reported [30], genus *Streptococcus* and order *Lactobacillales* were increased in patients taking PPI/H2 blockers, which inhibited stomach acid production. Anti-acid drugs might reduce the acid level and increase the survival rate of bacteria occurring mainly in the upper digestive tract, resulting in increased abundance in feces.

### Dietary habits

**Fig. 3:**
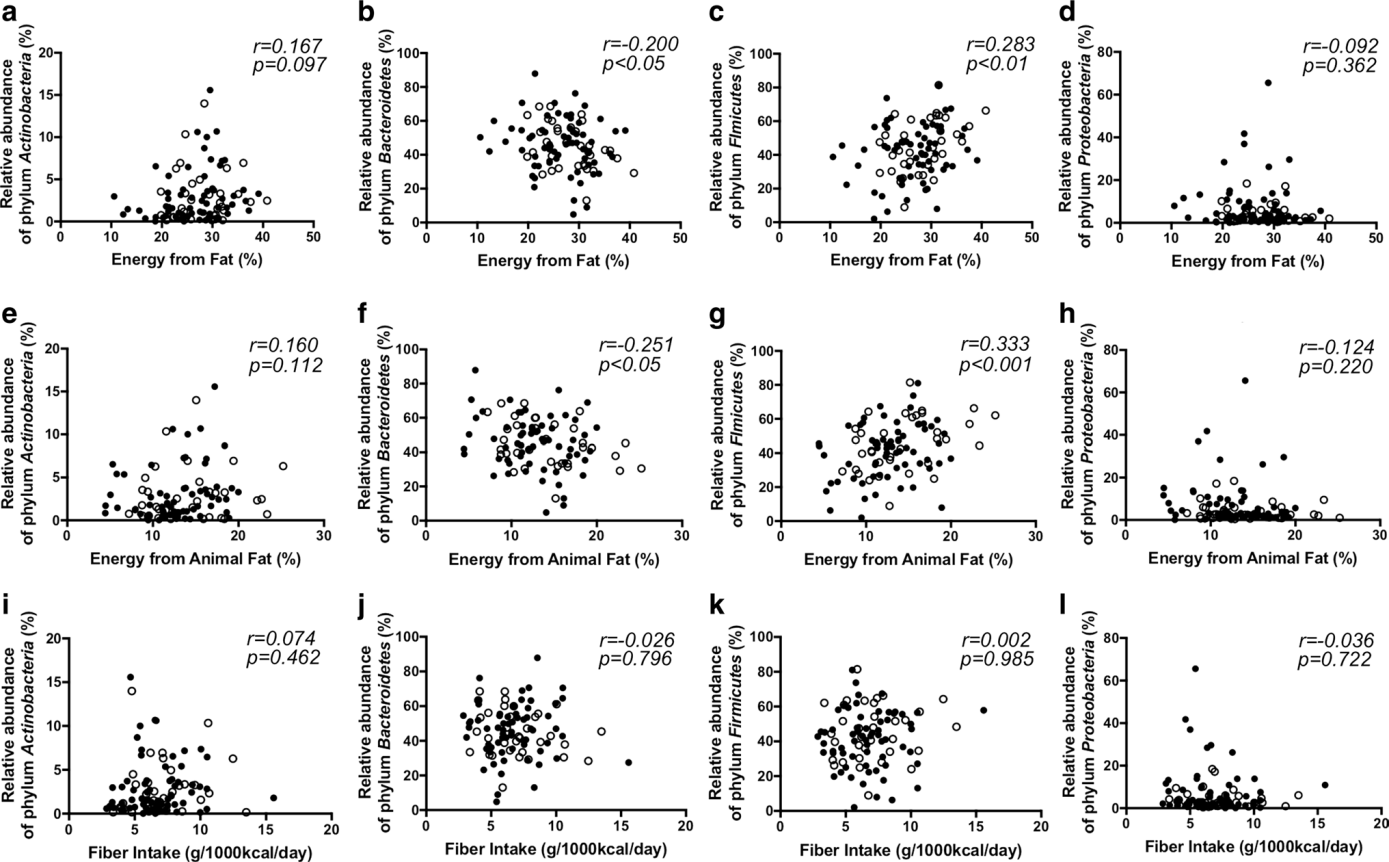
Gut microbial abundance of phylum level and dietary composition. **a**–**d** Correlation between the proportion of dietary fat in energy intake and relative abundance of the major phyla. **e**–**h** Correlation between the proportion of dietary animal fat in energy intake and relative abundance of the major phyla. **i**–**l** Correlation between the intake of dietary fiber per 1000 kcal and relative abundance of the major phyla

There were no significant differences in the intake of energy, water, electrolytes, or vitamins between the two groups, while AF patients tended to take more energy from animal fat compared to control subjects (Table 2). The correlation between fat intake and the major phyla *Actinobacteria, Bacteroidetes, Firmicutes,* and *Proteobacteria* (Fig. 3) showed that *Bacteroidetes* had a weak negative correlation with fat intake (*r* = − 0.20, *p* < 0.05) (especially animal fat [*r* = − 0.25, *p* < 0.05]), whereas *Firmicutes* showed the opposite association (fat: *r* = 0.28, *p* < 0.01, animal fat: *r* = 0.33, *p* < 0.001). Interestingly, the intake of non-specific n-3 PUFAs was higher in AF patients, although each type of n-3 PUFA was not significant (Table 2). The intake of C20:2 n-6 PUFAs (eicosadienoic acid), which are metabolized to arachidonic acid (AA), was significantly higher in AF patients (Supplemental Table 3).

**Table 2:** Dietary composition in control subjects vs. atrial fibrillation patients

	Control subjects (*n* = 66)	AF patients (*n* = 34)	*p* value
Energy (kcal)	1900 ± 65	2002 ± 75	0.21
Weight (g)	2384 ± 80	2430 ± 89	0.72
Water (g)	1978 ± 70	2001 ± 79	0.84
Energy from total protein (%)	15.3 ± 0.4	16.0 ± 0.5	0.23
Energy from animal protein (%)	8.8 ± 0.4	9.7 ± 0.5	0.15
Energy from vegetable protein (%)	6.2 (5.7–7.0)	6.3 (5.6–7.0)	0.92
Energy from total fat (%)	26.3 ± 0.7	28.3 ± 0.9	0.11
Energy from animal fat (%)	12.7 ± 0.5	14.3 ± 0.8	0.07
Energy from vegetable fat (%)	13.7 ± 0.5	14.0 ± 0.6	0.66
Energy from saturated fat (%)	7.1 ± 0.2	7.6 ± 0.3	0.28
Energy from carbohydrate (%)	51.1 ± 1.0	49.9 ± 1.6	0.48
Saturated fatty acid/1000 kcal (mg/1000 kcal)	7.9 ± 0.3	8.4 ± 0.4	0.28
n-3 PUFA/1000 kcal (mg/1000 kcal)	1.4 (1.1–1.8)	1.7 (1.3–2.0)	< 0.05
n-6 PUFA/1000 kcal (mg/1000 kcal)	5.5 (5.0–6.5)	5.8 (5.0–6.5)	0.39
n-6/3 ratio	4.0 ± 0.1	3.7 ± 0.2	0.13
Eicosadienoic acid/1000 kcal	25.3 ± 0.9	29.4 ± 1.4	< 0.05
Cholesterol/1000 kcal (mg/1000 kcal)	201.4 (160.8–256.7)	199.2 (166.2–250.8)	0.73
Total dietary fiber (g/1000 kcal)	6.6 (5.4–7.9)	6.6 (5.0–8.4)	0.58
Water soluble dietary fiber/1000 kcal (g/1000 kcal)	1.6 (1.3–2.0)	1.6 (1.3–2.1)	0.91
Insoluble dietary fiber/1000 kcal (g/1000 kcal)	4.6 (3.7–5.8)	4.8 (3.6–5.9)	0.71
Salt equivalent/1000 kcal (g/1000 kcal)	5.7 ± 0.2	5.8 ± 0.2	0.68
Sugar/1000 kcal (g/1000 kcal)	6.0 (3.6–8.1)	5.8 (3.4–7.5)	0.57
Alcohol/1000 kcal (g/1000 kcal)	4.9 (0.2–15.2)	1.6 (0–11.3)	0.10

Dietary composition for 1 month before stool sampling was assessed by using BDHQ. Values are expressed as mean ± standard deviation for normally distributed variables, and median and interquartile range for non-normally distributed variables

## Discussion

The relationship of CAD and HF to gut microbiota and its metabolites has been reported in cardiovascular diseases [3–7, 9]. AF has also been associated with gut microbial metabolites [8, 12, 13], although the alteration of gut microbial composition in AF is unclear.

Here, we measured the gut microbial composition in control subjects and AF patients. Unlike a previous report [20], gut microbial richness was lower in AF patients. Gut microbial composition changes with age, and gut microbial richness is no exception, increasing with age [31]. The difference in age between control and AF groups could have been a confounding factor in the previous report. To our knowledge, low bacterial richness is associated with insulin resistance, dyslipidemia, and inflammation, which are risk factors of AF [32]. Thus, a reduction in gut microbial richness may be associated with metabolic and chronic inflammatory diseases, including AF.

Enterotype 2, which is characterized by an abundance of the genus *Prevotella,* was decreased, while enterotype 3, which is characterized by an abundance of the genus *Ruminococcus*, was increased in AF. Previously [20], when participants were divided into two clusters, the AF group was less in enterotype 2, (dominated by *Prevotella*) but had more of enterotype 1 (dominated by *Bacteroides*). A lower *Prevotella*-prominent enterotype in AF patients was obtained in both studies. The increase in the *Ruminococcus-*predominant enterotype is characteristic of AF patients, and similar results were obtained from studies of CAD [3], symptomatic atherosclerosis [33], and obstructive sleep apnea–hypopnea syndrome (OSAHS) [34]. Patients with atherosclerosis and OSAHS have risk factors similar to those of AF patients. In OSAHS, hypoxia during sleep stimulates sympathetic nerve activity, which possibly triggers AF. OSAHS itself is a risk factor of AF [35]. Alteration of gut microbial enterotypes might be involved in the pathogenesis of these diseases.

In a previous report [20], the proportions of *Eubacterium*, *Roseburia*, *Ruminococcus*, *Blautia*, *Streptococcus*, *Dorea*, *Veillonella*, and *Enterococcus* (phylum *Firmicutes*) were much higher, while the proportions of *Prevotella* and *Alistipes* (phylum *Bacteroidetes*) and *Sutterella* and *Bilophila* (phylum *Proteobacteria*) were depleted in AF. The preponderance of genera belonging to *Proteobacteria* in control subjects and of the genera *Streptococcus* and *Dorea* in AF were similar to our results. It is noteworthy that patients with AF shared the enrichment of numerous microbial flora, such as *Streptococcus* and *Dorea*, demonstrated in hypertension [36], heart failure [37], and CAD [4]. An excess of these genera agrees with a recent study showing that overgrowth of genera is related to plasma and fecal indole [20]. Dietary tryptophan is converted to indole by the gut microbial enzyme tryptophanase. Indole is subsequently oxidized and sulfated in the host liver to form IS [9]. IS induces oxidative stress and consequently increases pulmonary vein and atrial arrhythmogenesis [13]. Koike H et al. demonstrated that the maintenance of sinus rhythm after AF ablation decreased serum IS levels in patients with high IS [38]. This suggests that serum IS may not only induce the onset of AF but may also be affected by the presence of AF. Another gut microbial metabolite, TMAO, could increase the instability of atrial electrophysiology [8]. *Lachnoclostridium*, *Parabacteroides*, and *Dorea,* which were increased in AF patients, produce high amounts of TMAO in the human gut [39]. Conversely, *Enterobacter*, which was depleted in AF patients, can consume TMAO [40]. The alteration of these gut microbiota might affect gut microbial metabolites and consequently, the pathogenesis of AF.

Hypertension is reported to have an association with gut microbiota [11]; increased abundance of *Prevotella* species (sp.), *Klebsiella* sp., and *Enterobacter* sp. has been observed in hypertension patients. Li et al. demonstrated elevation of blood pressure after fecal transplantation from hypertensive human donors to germ-free mice, compared with that after transplantation from control donors with normal blood pressure [11]. This suggested that the alteration of gut microbiota during hypertension was not the result of hypertension, but may be one of the causes of hypertension. Although the prevalence of HT was matched, the blood pressure was lower in AF patients than in control subjects. In this study, we demonstrated that the abundance of *Prevotella*-enterotype and *genus Enterobacter* was reduced in AF patients. These changes in gut microbiota were opposite to those observed in hypertension patients [11], and may account for the lowering of blood pressure in AF patients. Alteration of hemodynamics and reduction in cardiac output due to AF may affect the gut microbiota. However, there is no evidence to date linking the relationship between hemodynamics and gut microbiota. The relationship between the gut microbiota and heart failure, in which cardiac output may be reduced, has been reported [9, 37]. However, the results of those studies were contrasting, and the specific gut microbial phenotype of heart failure remains controversial. In this study, we were unable to clarify the relationship between hemodynamics and gut microbiota due to lack of data. Future studies should elucidate this association.

Contrary to a previous report on the correlation between dietary fat and gut microbial composition [41], there was a weak positive correlation between fat intake and *Firmicutes*, but not *Bacteroidetes*. We previously reported that *Bacteroides vulgatus* and *Bacteroides dorei* reduce plasma LPS activities and show anti-inflammatory functions. High-fat diet was found to induce metabolic endotoxemia and inflammation by increasing intestinal permeability [5]. An animal-based diet increased the abundance of *Alistipes*, *Parabacteroides*, and *Odoribacter,* which were increased with AF [42]. Fat intake may trigger AF via metabolic endotoxemia and chronic inflammation.

The significantly higher n-3 PUFA intake in AF patients does not contradict the report that the supplementation of fish oil (enriched in n-3 PUFAs) depleted members of the family *Enterobacteriaceae* [43]. Multiple randomized controlled trials have assessed the effects of n-3 PUFA supplementation on cardiovascular events [44], which have shown no benefits of n-3 PUFA supplementation for AF. A Danish cohort study showed a U-shaped association between the consumption of marine n-3 PUFA and AF risk, with the lowest risk close to the median intake of n-3 PUFA [45]. Although only the anti-inflammatory effects of n-3 PUFAs are attracting attention, the effect on gut microbiota should also be investigated. A high intake of n-6 PUFA is associated with inflammatory bowel disease, because most n-6 PUFAs are metabolized to AA, which is a precursor to potent pro-inflammatory mediators [46]. In AF patients, the intake of eicosadienoic acid, an n-6 PUFA, was increased. Eicosadienoic acid is converted to AA, modulates the production of pro-inflammatory modulators in macrophages, and is associated with prolonged inflammation [47]. Dietary composition is important to host immunity with or without alteration of gut microbial composition.

We should consider the impact of medication on the alteration of gut microbiota, especially PPI. Since most AF patients took PPI for the prevention of esophageal ulcers before ablation, we could not match PPI administration between control subjects and AF patients. Oral administration of PPI alters gut microbiota [30] and reduces its richness and diversity. PPI use reduces the intragastric acid concentration and impairs the bactericidal effect of pharyngeal bacteria. Consequently, the *Lactobacillales* order, and particularly genus *Streptococcus,* is more abundant in PPI users, as is *Parabacteroides*. It is unclear whether *Streptococcus* and *Parabacteroides* abundance in AF patients was affected by PPI.

The present study had several limitations. First, the number of patients was small; therefore, larger studies are warranted to verify our observations. Second, the residential locations of the AF patients differed from those of control subjects, and we cannot exclude the influence of regional differences. In addition, the absence of AF in control subjects was determined only through self-assessment questionnaires of anamnesis and drug uses; therefore, we could not exclude subclinical AF in control subjects. Finally, medication should be considered a confounding factor; however, we could not completely match the medication between the two groups.

Nevertheless, our results indicated that gut microbial composition was altered in AF patients. In addition, alteration of gut microbiota was possibly related to dietary composition. A prospective cohort study is needed to identify whether the alteration of gut microbiota involves the etiology of AF.

## Electronic supplementary material

Below is the link to the electronic supplementary material.

Supplementary file1 (PDF 59 kb)
