# Supplementary material for: Gut microbial composition in patients with atrial fibrillation: effects of diet and drugs
Source: Heart Vessels. 2020 Jul 18;36(1):105–14. doi: 10.1007/s00380-020-01669-y (PMC7788021; doi:10.1007/s00380-020-01669-y)
Supplement: Supplementary file 1 — Supplementary file1 (PDF 59 kb) [file 380_2020_1669_MOESM1_ESM.pdf]

Gut microbial composition in patients with atrial fibrillation: Effects of diet and drugs

Heart and vessels

Tokiko Tabata[1], Tomoya Yamashita[1], Koji Hosomi[2], Jonguk Park[3], Tomohiro Hayashi[1], Naofumi Yoshida[1], Yoshihiro Saito[1], Koji Fukuzawa[1], Kana Konishi[4], Haruka Murakami[4], Hitoshi Kawashima[3], Kenji Mizuguchi[3, 5], Motohiko Miyachi[4], Jun Kunisawa[2], Ken-ichi Hirata[1]

[1]Division of Cardiovascular Medicine, Department of Internal Medicine, Kobe University Graduate School of Medicine, Kobe, Japan

[2]Laboratory of Vaccine Materials and Laboratory of Gut Environmental System, National Institutes of Biomedical Innovation, Health and Nutrition (NIBIOHN), Osaka, Japan

[3]Laboratory of Bioinformatics, National Institutes of Biomedical Innovation, Health and Nutrition (NIBIOHN), Osaka, Japan

[4]Department of Physical Activity Research, National Institutes of Biomedical Innovation, Health and Nutrition (NIBIOHN), Tokyo, Japan

[5]Institute for Protein Research, Osaka University, Osaka, Japan

Corresponding author:

Tomoya Yamashita, M.D., Ph.D.

Division of Cardiovascular Medicine, Department of Internal Medicine

Kobe University Graduate School of Medicine

7-5-1 Kusunoki-cho, Chuo-ku, Kobe 6500017, Japan

Phone: +81-78-382-5111, Fax: +81-78-382-5859

E-mail: tomoya@med.kobe-u.ac.jp

Phylum level	Control (%)	AF (%)	p value	q value
Firmicutes	41.06 ± 2.01	45.60 ± 2.74	0.188	> 0.999
Verrucomicrobia	0.000 [0.000-0.043]	0.000 [0.000-0.135]	0.385	> 0.999
Proteobacteria	3.161 [1.434-8.882]	2.598 [1.456-5.893]	0.423	0.846
Bacteroidetes	45.65 ± 1.91	44.82 ± 2.29	0.793	> 0.999
Actinobacteria	1.692 [0.726-3.718]	1.988 [0.532-3.771]	0.864	> 0.999
Fusobacteria	0.000 [0.000-0.658]	0.000 [0.000-0.273]	0.933	0.933

Supplemental Table 1: Gut microbial abundance of phylum level control subjects vs AF patients
Values are expressed as mean±standard deviation for normally distributed variable, median and interquartile range for non-normally distributed variables.

Genus level	Control (%)	AF (%)	p value	q value
Enterobacter	0.052 [0.000-2.653]	0.000 [0.000-0.010]	< 0.001	0.008
Butyricimonas	0.026 [0.000-0.140]	0.165 [0.008-0.404]	0.001	0.053
Dorea	0.175 [0.018-0.329]	0.431 [0.104-0.644]	0.002	0.043
Lachnoclostridium	0.480 [0.305-0.950]	0.900 [0.534-1.476]	0.002	0.042
Lachnospiraceae_uncultured	0.390 [0.215-0.735]	0.737 [0.338-1.451]	0.003	0.049
Parabacteroides	1.121 [0.526-2.552]	2.229 [1.376-3.853]	0.005	0.060
Streptococcus	0.170 [0.023-0.872]	0.634 [0.110-2.349]	0.012	0.125
Odoribacter	0.121 [0.000-0.305]	0.238 [0.094-0.527]	0.012	0.111
Barnesiella	0.021 [0.000-0.208]	0.278 [0.000-1.078]	0.015	0.128
Flavonifractor	0.041 [0.011-0.113]	0.098 [0.021-0.179]	0.019	0.146
Veillonella	0.026 [0.000-0.144]	0.084 [0.018-0.413]	0.022	0.149
Alistipes	0.360 [0.090-1.420]	1.050 [0.200-3.010]	0.024	0.150
Catenibacterium	0.000 [0.000-0.000]	0.000 [0.000-0.141]	0.031	0.182
Paraprevotella	0.000 [0.000-0.000]	0.000 [0.000-0.665]	0.033	0.181
Megamonas	0.000 [0.000-0.010]	0.000 [0.000-0.261]	0.070	0.354
Prevotellaceae_uncultured	0.000 [0.000-0.000]	0.000 [0.000-0.000]	0.117	0.556
Sutterella	0.507 [0.000-1.551]	0.956 [0.106-2.098]	0.125	0.559
Ruminococcus 1	0.016 [0.000-0.419]	0.101 [0.000-1.511]	0.125	0.529
Eubacterium ruminantium group	0.000 [0.000-0.000]	0.000 [0.000-0.000]	0.139	0.558
Dialister	0.000 [0.000-0.024]	0.000 [0.000-0.079]	0.151	0.545
Prevotellaceae NK3B31 group	0.000 [0.000-0.000]	0.000 [0.000-0.335]	0.171	0.590
Prevotellaceae UCG-003	0.000 [0.000-0.000]	0.000 [0.000-0.000]	0.174	0.573
Parasutterella	0.052 [0.000-0.518]	0.021 [0.000-0.190]	0.180	0.569
Holdemanella	0.000 [0.000-0.328]	0.000 [0.000-0.003]	0.238	0.724
Eubacterium ventriosum group	0.053 [0.000-0.203]	0.089 [0.010-0.238]	0.241	0.705
Clostridium sensu stricto 1	0.000 [0.000-0.069]	0.010 [0.000-0.231]	0.253	0.713
Akkermansia	0.000 [0.000-0.043]	0.000 [0.000-0.140]	0.293	0.796
Eubacterium eligens group	0.005 [0.000-0.137]	0.059 [0.000-0.377]	0.295	0.774
Lachnospiraceae NK4A136 group	0.145 [0.031-0.562]	0.250 [0.028-0.612]	0.298	0.755
Ruminococcus torques group	0.811 [0.383-1.828]	0.943 [0.540-2.225]	0.319	0.782
Ruminococcaceae UCG-002	0.042 [0.000-0.792]	0.166 [0.000-1.507]	0.326	0.774
Lachnospira	0.366 [0.018-1.111]	0.662 [0.101-1.315]	0.340	0.783
Ruminiclostridium 5	0.219 [0.099-0.559]	0.181 [0.088-0.506]	0.348	0.777
Erysipelotrichaceae UCG-003	0.000 [0.000-0.101]	0.000 [0.000-0.126]	0.365	0.792
Klebsiella	0.000 [0.000-0.100]	0.010 [0.000-0.140]	0.375	0.792
Lactobacillus	0.000 [0.000-0.021]	0.000 [0.000-0.133]	0.391	0.803
Phascolarctobacterium	0.690 [0.201-1.255]	0.865 [0.053-1.822]	0.448	0.896
Lachnospiraceae UCG-004	0.136 [0.051-0.263]	0.084 [0.041-0.249]	0.490	0.955
Ruminiclostridium 9	0.052 [0.021-0.182]	0.051 [0.022-0.117]	0.512	0.972
Ruminococcaceae_uncultured	0.270 [0.105-0.707]	0.409 [0.158-0.865]	0.516	0.956
Coprococcus 1	0.095 [0.000-0.172]	0.092 [0.000-0.282]	0.528	0.955
Coprococcus 2	0.000 [0.000-0.000]	0.000 [0.000-0.000]	0.542	0.959
Christensenellaceae R-7 group	0.011 [0.000-0.183]	0.037 [0.000-0.418]	0.591	> 0.999

Ruminococcaceae UCG-003	0.026 [0.000-0.230]	0.073 [0.000-0.291]	0.597	> 0.999
Fusicatenibacter	0.386 [0.115-0.940]	0.438 [0.101-1.291]	0.601	0.992
Ruminococcaceae NK4A214 group	0.000 [0.000-0.223]	0.010 [0.000-0.164]	0.613	0.991
Prevotella 2	0.000 [0.000-0.010]	0.000 [0.000-0.003]	0.629	0.995
Eubacterium hallii group	0.815 [0.313-1.353]	0.713 [0.411-1.192]	0.634	0.983
Lachnospiraceae UCG-008	0.180 [0.050-0.414]	0.177 [0.082-0.342]	0.637	0.967
Bifidobacterium	1.265 [0.304-2.972]	1.030 [0.010-2.955]	0.661	0.985
Eubacterium coprostanoligenes group	0.214 [0.000-0.750]	0.228 [0.000-0.760]	0.662	0.967
Collinsella	0.324 [0.057-0l755]	0.380 [0.068-0.868]	0.667	0.957
Roseburia	2.378 [0.718-4.365]	2.179 [1.323-3.440]	0.692	0.974
Blautia	2.735 [1.478-5.270]	2.835 [1.183-5.570]	0.715	0.987
Faecalibacterium	7.376 [1.676-12.81]	5.269 [1.759-12.28]	0.715	0.970
Anaerostipes	0.439 [0.198-1.368]	0.536 [0.130-1.140]	0.716	0.955
Ruminococcaceae UCG-005	0.016 [0.000-0.252]	0.036 [0.000-0.280]	0.722	0.946
Bacteroidales S24-7 group_uncultured bacterium	0.020 [0.000-0.198]	0.016 [0.000-0.037]	0.728	0.938
Bacteroides	29.30 [14.84-49.23]	30.47 [18.83-38.08]	0.753	0.954
Anaerotruncus	0.064 [0.000-0.179]	0.052 [0.000-0.254]	0.768	0.956
Butyricicoccus	0.426 [0.134-0.709]	0.372 [0.174-0.599]	0.781	0.957
Ruminococcaceae UCG-013	0.201 [0.000-0.416]	0.162 [0.011-0.480]	0.798	0.962
Ruminococcus 2	0.000 [0.000-0.823]	0.000 [0.000-0.888]	0.798	0.948
Ruminococcaceae UCG-014	0.000 [0.000-0.034]	0.000 [0.000-0.039]	0.839	0.981
Peptoclostridium	0.031 [0.010-0.094]	0.026 [0.008-0.119]	0.881	> 0.999
Prevotella 9	0.000 [0.000-3.050]	0.000 [0.000-0.035]	0.895	> 0.999
Ruminiclostridium 6	0.000 [0.000-0.042]	0.000 [0.000-0.026]	0.895	> 0.999
Megasphaera	0.000 [0.000-0.067]	0.000 [0.000-0.172]	0.913	> 0.999
Alloprevotella	0.000 [0.000-0.000]	0.000 [0.000-0.000]	0.953	> 0.999
Subdoligranulum	0.980 [0.104-2.385]	0.541 [0.063-2.861]	0.962	> 0.999
Acidaminococcus	0.000 [0.000-0.096]	0.000 [0.000-0.118]	0.970	> 0.999
Bilophila	0.076 [0.008-0.272]	0.082 [0.001-0.218]	0.974	> 0.999
Fusobacterium	0.000 [0.000-0.672]	0.000 [0.000-0.296]	0.977	> 0.999
Escherichia-Shigella	0.068 [0.011-0.549]	0.071 [0.018-0.501]	0.986	0.999
Succinivibrio	0.000 [0.000-0.000]	0.000 [0.000-0.000]	> 0.999	> 0.999

Supplemental Table 2: Gut microbial abundance of genus level control subjects vs AF patients
Values are expressed as median and interquartile range for non-normally distributed variables.

	Control Subjects (n=66)	AF patients (n=34)	p value
n-3 PUFAs			
α Linolenic acid/1000 kcal	860.6 ± 32.4	935.2 ± 40.7	0.17
Stearidonic acid/1000 kcal	39.3 (27.7 -69.5)	60.8 (31.8 - 79.2)	0.13
Eicosatetraenoic acid/1000 kcal	14.8 (10.2 - 25.3)	22.9 (10.7 - 29.2)	0.17
Eicosapentaenoic acid/1000 kcal	144.5 (99.9 - 260.7)	229.7 (108.0 - 291.4)	0.17
Docosapentaenoic acid/1000 kcal	44.1 (33.4 - 74.6)	64.3 (33.7 - 80.6)	0.20
Docosahexaenoic acid/1000 kcal	251.5 (178.6 - 413.9)	356.0 (190.1 - 463.6)	0.19
n-6 PUFAs			
Linolenic acid/1000 kcal	5546 ± 174	5682 ± 191	0.40
γ Linolenic acid/1000 kcal	3.4 (2.2 - 5.1)	4.2 (2.6 - 6.7)	0.08
Eicosadienoic acid/1000 kcal	25.3 ± 0.9	29.4 ± 1.4	<0.05
Dihomo-γ Linolecic acid/1000 kcal	16.9 ± 0.6	18.5 ± 1.0	0.13
Arachidonic acid/1000 kcal	95.7 (75.8 - 116.9)	99.2 (77.8 - 116.1)	0.50

Supplemental Table 3: Dietary composition about polyunsaturated fatty acids control subjects vs AF patients Values are expressed as mean±standard deviation for normally distributed variable, median and interquartile range for non-normally distributed variables.
